# NuSegDA: Domain adaptation for nuclei segmentation

**DOI:** 10.3389/fdata.2023.1108659

**Published:** 2023-03-02

**Authors:** Mohammad Minhazul Haq, Hehuan Ma, Junzhou Huang

**Affiliations:** Department of Computer Science and Engineering, University of Texas at Arlington, Arlington, TX, United States

**Keywords:** nuclei segmentation, domain adaptation, Unsupervised Domain Adaptation, Semi-Supervised Domain Adaptation, adversarial learning

## Abstract

The accurate segmentation of nuclei is crucial for cancer diagnosis and further clinical treatments. To successfully train a nuclei segmentation network in a fully-supervised manner for a particular type of organ or cancer, we need the dataset with ground-truth annotations. However, such well-annotated nuclei segmentation datasets are highly rare, and manually labeling an unannotated dataset is an expensive, time-consuming, and tedious process. Consequently, we require to discover a way for training the nuclei segmentation network with unlabeled dataset. In this paper, we propose a model named NuSegUDA for nuclei segmentation on the unlabeled dataset (target domain). It is achieved by applying Unsupervised Domain Adaptation (UDA) technique with the help of another labeled dataset (source domain) that may come from different type of organ, cancer, or source. We apply UDA technique at both of feature space and output space. We additionally utilize a reconstruction network and incorporate adversarial learning into it so that the source-domain images can be accurately translated to the target-domain for further training of the segmentation network. We validate our proposed NuSegUDA on two public nuclei segmentation datasets, and obtain significant improvement as compared with the baseline methods. Extensive experiments also verify the contribution of newly proposed image reconstruction adversarial loss, and target-translated source supervised loss to the performance boost of NuSegUDA. Finally, considering the scenario when we have a small number of annotations available from the target domain, we extend our work and propose NuSegSSDA, a Semi-Supervised Domain Adaptation (SSDA) based approach.

## 1. Introduction

**Figure 1:**
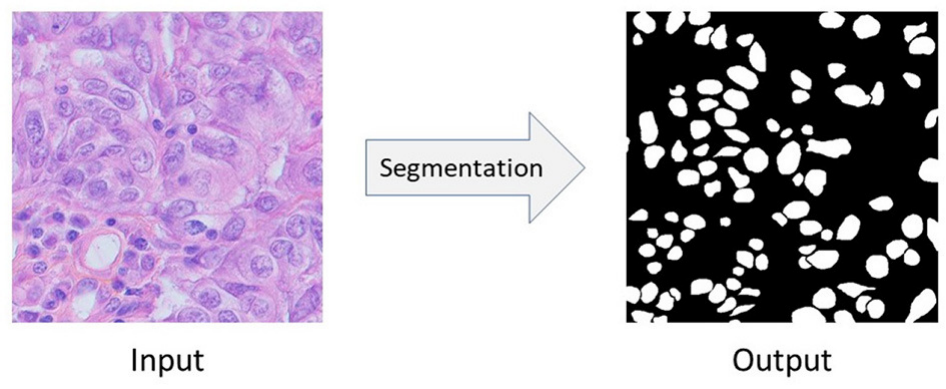
The semantic segmentation of nuclei. In this figure, the input image comes from Triple Negative Breast Cancer (TNBC).

Nuclei are the fundamental organizational unit of life (Sharma et al., 2022). Nuclei segmentation, a subclass of biomedical image segmentation, is considered as an essential task of digital histopathology image analysis (Yang S. et al., 2021; Haq and Huang, 2022). However, accurate nuclei segmentation is quite challenging due to the significant variations in the shape and appearance of nuclei, clustered and overlapped nuclei, blurred nuclei boundaries, inconsistent staining methods, scanning artifacts, etc. (see Figure 1). Also, histopathology of different organs or cancer types may exhibit different textures, color distributions, morphology, and scales (Xu et al., 2017; Mahmood et al., 2019).

Nuclei segmentation problem can be seen as a semantic segmentation problem in which we want to segment the nuclei from it's background. Figure 1 shows the input image, and corresponding output of semantic segmentation of nuclei. Convolutional Neural Network (CNN) based approaches like Fully Convolutional Network (FCN) (Long et al., 2015), U-Net (Ronneberger et al., 2015), UNet++ (Zhou et al., 2018), etc. give very promising results in biomedical image segmentation tasks as well as in nuclei segmentation problems (Sirinukunwattana et al., 2016; Haq and Huang, 2022; Sharma et al., 2022). However, to successfully train these fully-supervised methods, we need at least a few amount of annotated data (i.e., images with their corresponding pixel-level ground-truth labels) (Zeiler and Fergus, 2014; Kumar et al., 2017; Sharma et al., 2022). Unfortunately, such well-annotated datasets, even if very small-sized, are highly rare in biomedical domain. Moreover, due to the heterogeneity of nuclei, it's even harder to learn good models under the scenario of lacking annotations and samples. Also, commonly used strategy which first collects an unannotated histopathology dataset and then do the manual pixel-level labeling with the help of experts is also an expensive, time-consuming, and tedious process (Xu et al., 2017; Chen C. et al., 2019; Yang S. et al., 2021). For example, annotating even a small nuclei segmentation dataset consisting of 50 image patches takes 120–130 h of an expert pathologist's time (Hou et al., 2019). Therefore, an urgent question is raised: how could we robustly train a deep CNN model for nuclei segmentation without any further need for annotations?

For nuclei segmentation problem, simply applying Transfer Learning (i.e., models trained with one organ or cancer type, and then evaluated with different organ or cancer types) unfortunately leads to poor performance due to the domain shift problem (Sharma et al., 2022). This domain shift problem happens due to different scanners, scanning protocols, tissue types, etc. (Sharma et al., 2022). In this paper, we propose Domain Adaptation, a subclass of Transfer Learning, based framework to solve the domain shift problem for nuclei segmentation. We consider the unannotated dataset (i.e., for which we want to predict the labels) as the target domain. Then, with the help of another related but different annotated dataset, referred as the source domain, we apply adversarial learning (Goodfellow et al., 2014) based domain adaptation technique for nuclei segmentation problem. Thus, our proposed framework, learns from the labeled source domain and adapts to the unlabeled target domain.

**Figure 2:**
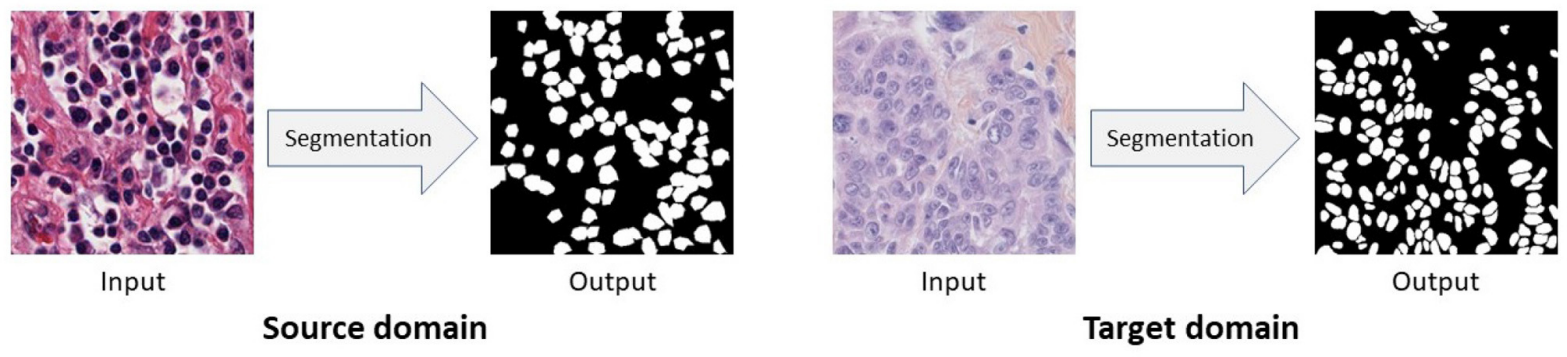
Images from different domains look dissimilar while their pixel-level segmentation outputs are similar. In this figure, source domain and target domain images come from Kidney Renal Clear cell carcinoma (KIRC) and Triple Negative Breast Cancer (TNBC), respectively.

In this work, we first propose an Unsupervised Domain Adaptation (UDA) model for nuclei segmentation to close the gap between the annotated source domain and unlabeled target domain. Unsupervised Domain Adaptation methods are capable to minimize the labeling cost by utilizing cross-domain data and aligning the distribution shift between labeled source domain data and unlabeled target domain data. We empirically and carefully observed that, images from different nuclei datasets, even if collected from different organ or cancer types, exhibit dissimilarity although their corresponding segmentation ground-truth labels are quite similar (see Figure 2). In summary, ground-truth labels for nuclei segmentation are domain-invariant. Because of the aforementioned observation, we apply domain adaptation in the output space. Thus, with the help of adversarial learning, we train a robust nuclei segmentation network to generate source-domain look-alike outputs for target images. Adversarial learning attempts to align target-domain predictions with source-domain ground truths via discriminator training. In addition to image-level domain adaptation at the output space, we apply domain-invariant class-conditional feature-level domain adaptation in the feature space. However, simply forcing the target-domain distribution toward the source-domain distribution can destroy the latent structural patterns of the target domain, leading to a drop in the model's accuracy. Consequently, we also use a reconstruction network to maximize the correlation between target images and target predictions. Again, a reconstruction network alone can not perfectly reconstruct original images (i.e., the reconstructed images lack original texture, style, color distribution, etc.) for which we incorporate adversarial learning into the reconstruction network, which in turn helps us to translate source domain images to the target domain. We additionally train our UDA model with these target-translated source images, and observe a significant performance boost. Finally, we extend our UDA framework to Semi-Supervised Domain Adaptation (SSDA) model considering that we have some annotations available from the target domain.

Conducting extensive experiments on two nuclei segmentation datasets we conclude that, our proposed UDA method, NuSegUDA, outperforms fully-supervised model trained on source domain and evaluated on target domain, and baseline generic and biomedical UDA segmentation models. Experimental result (see Section 4) also shows the impacts of training NuSegUDA with proposed image reconstruction adversarial loss, target-translated source images, and feature-level clustering loss. Furthermore, the accuracy of our SSDA model, NuSegSSDA, is highly competitive to the upper bound of fully-supervised model trained in the target domain.

Therefore, the main contributions of this paper are: (1) We propose an adversarial learning based Unsupervised Domain Adaptation (UDA) approach, which is applied at both of feature space and output space to solve nuclei segmentation problem for unannotated datasets. (2) Additionally, we incorporate adversarial learning into a reconstruction network to translate source domain images to the target domain, and train proposed model with these target-translated source images. (3) Compared to many of the baselines, our proposed method is simple as it does not depend on any data synthesization or data augmentation. (4) Our proposed UDA framework can be easily extended to Semi-Supervised Domain Adaptation (SSDA) in the scenario where a small portion of the target domain is labeled. (5) Extensive and comprehensive experiments on two datasets have demonstrated the superiority of the proposed methods.

## 2. Related works

In literature, several domain adaptation models have been proposed for generic image segmentation. Isola et al. (2017) applied conditional GAN (Mirza and Osindero, 2014) for image-to-image translation problems. CyCADA proposed an Unsupervised Domain Adaptation (UDA) model utilizing both of input space and feature space adaptation (Hoffman et al., 2017). A multi-level adversarial network based domain adaptation approach for semantic segmentation was proposed in AdaptSegNet (Tsai et al., 2018). Zhang et al. (2018) proposed a fully convolutional adaptation network for semantic segmentation. CrDoCo proposed a cross-domain consistency loss based pixel-wise adversarial domain adaptation algorithm (Chen Y.-C. et al., 2019). Yang J. et al. (2021) proposed adversarial self-supervision UDA model which maximizes agreement between clean samples and their adversarial examples. Toldo et al. (2021) proposed feature-clustering based UDA framework that groups features of the same class into tight and well-separated clusters.

Domain adaptation has also been employed in different biomedical image segmentation tasks. A multi-connected domain discriminator based UDA model for brain lesion segmentation was proposed by Kamnitsas et al. (2017). Dong et al. (2018) introduced another UDA framework for cardiothoracic ratio estimation through chest organ segmentation. Huo et al. (2018) proposed an end-to-end CycleGAN (Zhu et al., 2017) based whole abdomen MRI to CT image synthesis and CT splenomegaly segmentation network. Mahmood et al. (2019) proposed a nuclei segmentation approach in which a large dataset is generated using synthesization. Gholami et al. (2019) proposed a biophysics-based medical image segmentation framework which enriches the training dataset by generating synthetic tumor-bearing MR images. Hou et al. (2019) also synthesized annotated training data for histopathology image segmentation. Haq and Huang (2020) utilized adversarial learning at output space along with a reconstruction network for nuclei segmentation. Xia et al. (2020) proposed Uncertainty-aware Multi-view Co-Training (UMCT) framework which is capable of utilizing large-scale unlabeled data to improve volumetric medical image segmentation. Raju et al. (2020) proposed an user-guided domain adaptation framework for liver segmentation which uses prediction-based adversarial domain adaptation to model the combined distribution of user interactions and mask predictions. EndoUDA proposed another UDA-based segmentation model for gastrointestinal endoscopy imaging which comprises of a shared encoder and a joint loss function for improved unseen target domain generalization (Celik et al., 2021). Li et al. (2021) proposed another GAN (Mirza and Osindero, 2014) based framework for Unsupervised Domain Adaptation of nuclei segmentation which also utilized self-ensembling and conditional random field (Boykov and Kolmogorov, 2004). Sharma et al. (2022) proposed a mutual information based UDA method for cross-domain nuclei segmentation.

Several previous approaches (Dong et al., 2018; Tsai et al., 2018; Haq and Huang, 2020; Toldo et al., 2021) employed Unsupervised Domain Adaptation technique either in the output space or the feature space. Differently from these approaches, in our work we apply domain adaptation at both of output space and feature space. Additionally, unlike previous works, we utilize a reconstruction network to ensure that the target domain predictions spatially correspond to the target domain images. Also, several recent works (Huo et al., 2018; Gholami et al., 2019; Hou et al., 2019; Mahmood et al., 2019) applied complicated data synthesization techniques to generate a large training dataset. On the contrary, in our work we simply incorporate adversarial learning so that the source domain images can be translated to the target domain for further training.

## 3. Methodology

In this section, we first describe the problem that we aim to solve. Then, we introduce the details of our proposed Unsupervised Domain Adaptation (UDA) and Semi-Supervised Domain Adaptation (SSDA) framework. Finally, we discuss the implementations of the proposed models.

### 3.1. Problem definition

In our nuclei segmentation problem, we have nuclei histopathology image patches as input *X* of size *H*×*W*×3. The input *X* comes from either the source domain or the target domain. Depending on the problem (i.e., unsupervised or semi-supervised) and domain (i.e., source or target), we may also have the corresponding pixel-wise ground-truth label *Y* of size *H*×*W*×1 which is basically a binary mask. Then, using the segmentation network, we want to predict the segmentation output Ŷ of size *H*×*W*×1.

Formally, in Unsupervised Domain Adaptation (UDA) problem, the source domain consists of *N*_*s*_ annotated images {(*X*_*s*_, *Y*_*s*_)}, and the target domain has *N*_*t*_ unannotated images {(*X*_*t*_)}. In the case of Semi-Supervised Domain Adaptation (SSDA) problem, the source domain is the same as it is in UDA problem, and we assume that the target domain has $N_{t}^{l}$ images with annotations $\left\{\left(X_{t}^{l},Y_{t}\right)\right\}$ and $N_{t}^{u}$ unannotated images $\left\{\left(X_{t}^{u}\right)\right\}$. In both of UDA and SSDA problem, the source domain data and target domain data are the related data but they come from different distributions (i.e., different organ or cancer types). For both of unsupervised and Semi-Supervised Domain Adaptation, our ultimate goal is to learn nuclei segmentation models that accurately produce the segmentation outputs in the target domain.

### 3.2. Unsupervised Domain Adaptation

**Figure 3:**
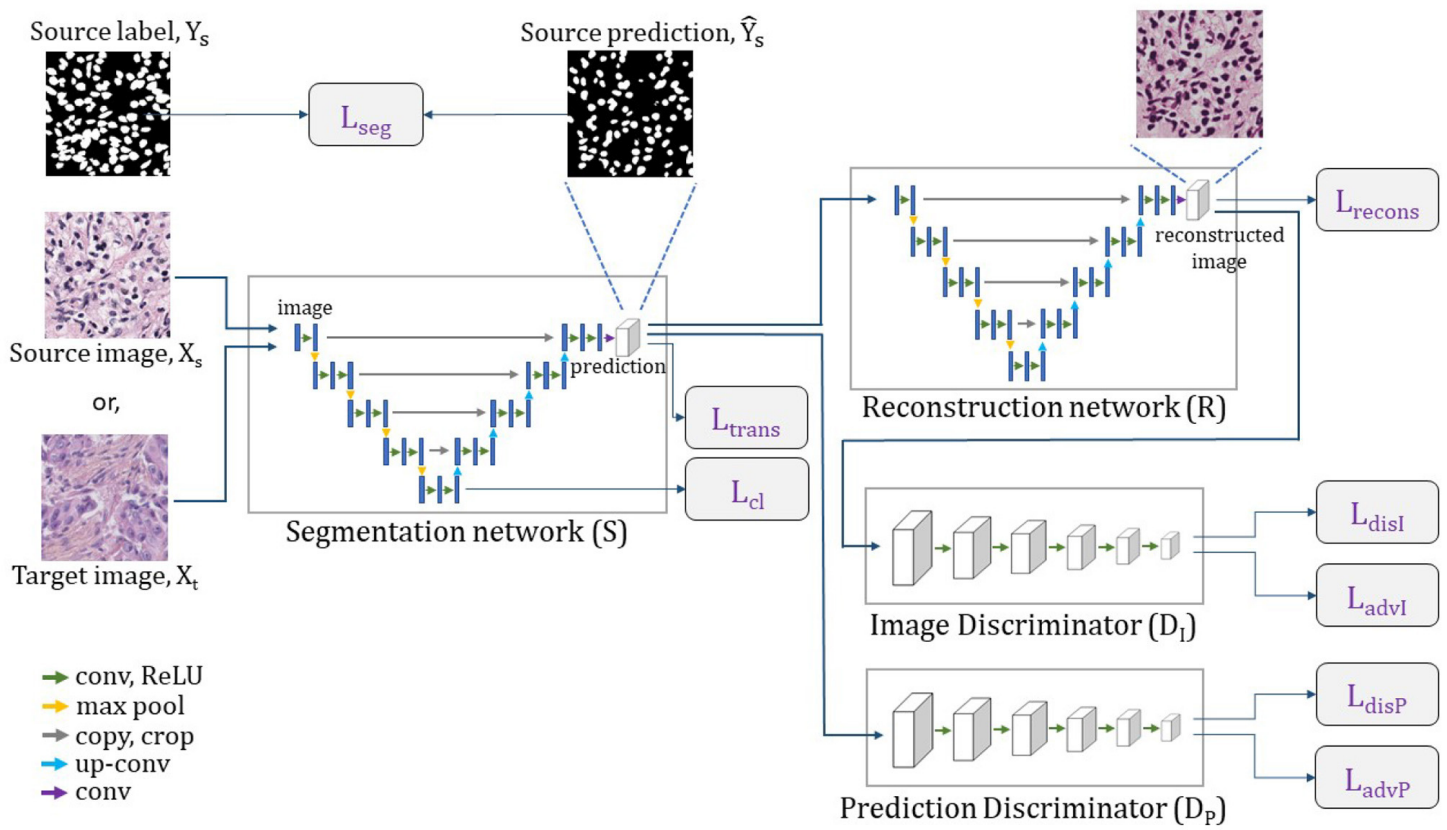
Complete architecture of NuSegUDA. Segmentation network generates segmentation outputs, from which reconstruction network reconstructs input images. Prediction discriminator distinguishes between source domain outputs and target domain outputs. Image discriminator distinguishes between original images and reconstructed images.

We refer our nuclei segmentation Unsupervised Domain Adaptation (UDA) model as NuSegUDA, and the framework is shown in Figure 3. NuSegUDA consists of four modules: Segmentation network (*S*), Reconstruction network (R), Prediction Discriminator (*D*_*P*_), and Image Discriminator (*D*_*I*_).

#### 3.2.1. Segmentation network

The segmentation network *S* takes image *X* as the input and produces the segmentation prediction Ŷ of the same size as the input. Here, *X* can be either the source domain image *X*_*s*_, or the target domain image *X*_*t*_. Hence, the source domain prediction $\hat{Y_{s}}=S\left(X_{s}\right)$, and the target domain prediction $\hat{Y_{t}}=S\left(X_{t}\right)$. From the perspective of GAN (Goodfellow et al., 2014) framework, the segmentation network *S* can be thought as the generator module.

We train *S* to generate the source domain segmentation predictions $\hat{Y_{s}}$ to be similar to the source domain ground-truth labels *Y*_*s*_. Since in Unsupervised Domain Adaptation (UDA) the ground-truth labels are not available for target images, we can not compute any supervised pixel-level loss for target predictions. In practice, we found that combining dice-coefficient loss and entropy minimization loss is more effective than simply using binary cross-entropy loss for nuclei segmentation tasks. Therefore, we define segmentation loss *L*_*seg*_ as:


(1) $$\begin{array}{l} L_{dice}\left(X_{s}\right)=1-\frac{2.Y_{s}^{\prime }.\hat{Y_{s}^{\prime }}}{Y_{s}^{\prime }+\hat{Y_{s}^{\prime }}} \end{array}$$

(2) $$\begin{array}{l} L_{em}\left(X_{s}\right)=-\frac{1}{H\times W}\underset{h,w}{\sum }\hat{Y_{s}}\log\left(\hat{Y_{s}}\right) \end{array}$$

(3) $$\begin{array}{l} L_{seg}\left(X_{s}\right)=L_{dice}\left(X_{s}\right)+L_{em}\left(X_{s}\right) \end{array}$$

where $Y_{s}^{\prime }$ and $\hat{Y_{s}^{\prime }}$ are the flattened *Y*_*s*_ and $\hat{Y_{s}}$, respectively.

Here, question may arise that why we are using single segmentation network *S* in NuSegUDA although we have two different domains. Since we are particularly looking for nuclei from both domain images, it is very unusual to use multiple segmentation networks. Additionally, using two segmentation networks would increase the number of learnable parameters which would slow down the training process in turn. Therefore, single segmentation network helps to prevent the memory issues and training latency in NuSegUDA.

Training the segmentation network *S* with only the annotated source data teaches *S* to make accurate predictions for source images. However, this segmentation network may generate incorrect outputs for target images as there are visual discrepancies between source images and target images (see Figure 2). This visual gap between domains causes the domain shift problem. According to our aforementioned observation that nuclei segmentation outputs are domain-invariant, we require *S* to produce target domain predictions as much as close to the source domain predictions. In other words, we want to make the distribution of target predictions $\hat{Y_{t}}$ closer to the distribution of source predictions $\hat{Y_{s}}$. For this reason, we utilize Prediction Discriminator *D*_*P*_ in NuSegUDA, and we define the prediction adversarial loss as:


(4) $$\begin{array}{l} L_{advP}\left(X_{t}\right)=-\frac{1}{H_{p}\times W_{p}}\underset{h_{p},w_{p}}{\sum }\log\left(D_{P}\left({Ŷ}_{t}\right)\right) \end{array}$$

where Ŷ_*t*_ = *S*(*X*_*t*_), and *H*_*p*_ and *W*_*p*_ are height and width of the prediction discriminator output *D*_*P*_(Ŷ_*t*_). The details of the Prediction Discriminator *D*_*P*_ is discussed in Section 3.2.3.

The prediction adversarial loss in Equation (4) helps *S* to fool the prediction discriminator so that it considers $\hat{Y_{t}}$ as source domain segmentation outputs. Segmentation loss and the prediction adversarial loss jointly guide *S* to generate target domain predictions Ŷ_*t*_ which look similar to source domain ground-truths.

#### 3.2.2. Reconstruction network

As we mentioned earlier, the segmentation network *S* produces domain-invariant predictions for both domains. In other words, we want to generate the target domain predictions in a way so that they become similar to the source domain predictions. However, it is highly probable that the target predictions are not well-correlated with corresponding target input images. In this scenario, the ability of reconstructing the images from the predictions with similar visual appearance as input images will ensure that there is a correlation between the input image and segmentation output.

To ensure that our target domain predictions spatially correspond to the target domain images, reconstruction network *R* is used in NuSegUDA. In a similar way to Xia and Kulis (2017), we consider the segmentation network *S* and the reconstruction network *R* as an encoder and a decoder, respectively. *R* reconstructs target images from the corresponding predictions. Thus, *S* and *R* altogether works as an autoencoder.

Using our reconstruction network *R*, we first reconstruct target input images *X*_*t*_ from Ŷ_*t*_. Then, we calculate the reconstruction loss as:


(5) $$\begin{array}{l} L_{recons}\left(X_{t}\right)=\frac{1}{H\times W\times C}\underset{h,w,c}{\sum }{\left(X_{t}-R\left({Ŷ}_{t}\right)\right)}^{2} \end{array}$$

where, *R*(Ŷ_*t*_) is the output of reconstruction network for Ŷ_*t*_, and *C* is the number of channels of input image *X*_*t*_.

**Figure 4:**
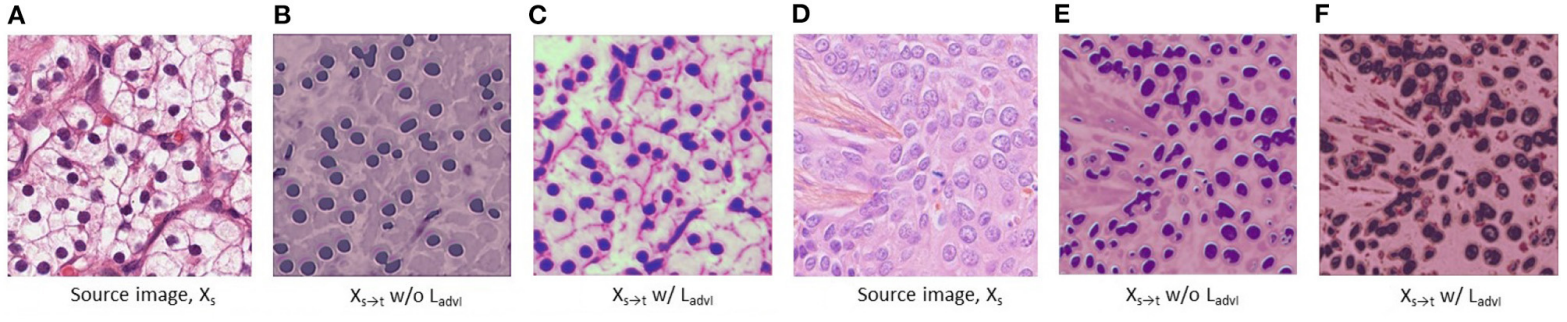
Visualization of the target-translated source domain images *X*_*s*→*t*_ which are also the same as reconstructed source images $\tilde{X_{s}}$. **(A–C)** and **(D–F)** are chosen from Kidney Renal Clear cell carcinoma (KIRC) domain, and Triple Negative Breast Cancer (TNBC) domain, respectively. In **(C)** and **(F)**, we see that KIRC domain image is translated (i.e., reconstructed) into TNBC domain styles, and vice versa, respectively. In **(B, C)** and **(E, F)**, *X*_*s*→*t*_ w/o *L*_*advI*_ and *X*_*s*→*t*_ w/ *L*_*advI*_ refer to the translated image when NuSegUDA is trained without and with image reconstruction adversarial loss *L*_*advI*_, respectively.

Although we use above reconstruction loss to reconstruct the target domain images from its predictions, the reconstructed images may have very different textures and styles (for both of nuclei and background) than the original images (see Figure 4). The reason is that the pixel-wise reconstruction loss *L*_*recons*_ (in Equation 5) can not capture the overall pixel distribution of target domain images. To solve this issue, in addition to *L*_*recons*_, we also utilize an Image Discriminator *D*_*I*_ to distinguish the original images and the reconstructed images. To train *R* and *S* to generate original-alike reconstructed images, we define image reconstruction adversarial loss as:


(6) $$\begin{array}{l} L_{advI}\left(X_{t}\right)=-\frac{1}{H_{i}\times W_{i}}\underset{h_{i},w_{i}}{\sum }\log\left(D_{I}\left(\tilde{X_{t}}\right)\right) \end{array}$$

where $\tilde{X_{t}}=R\left({Ŷ}_{t}\right)$, and *H*_*i*_ and *W*_*i*_ are height and width of the image discriminator output $D_{I}\left(\tilde{X_{t}}\right)$. This adversarial loss *L*_*advI*_ trains *R* and *S* to reconstruct target domain images of similar distributions (in terms of texture, style, color distribution, etc.) to the original images from target domain.

In NuSegUDA, *L*_*advP*_ helps the segmentation network *S* to generate target predictions $\hat{Y_{t}}$ to be similar to the source predictions $\hat{Y_{s}}$. And, due to *L*_*advI*_, reconstruction network *R* learns to reconstruct target images (i.e., $\tilde{X_{t}}$) which are very similar to the original target images in terms of texture, style, color distribution, etc. In other words, *S* maps both domain images (i.e., *X*_*s*_ and *X*_*t*_) to a common prediction subspace ${\mathbb{R}}_{p}^{n}$, and from ${\mathbb{R}}_{p}^{n}$ *R* reconstructs the images in target domain. Therefore, using *S* and *R* we can translate source domain images *X*_*s*_ to the target domain. Thus, target translated source domain images *X*_*s*→*t*_ = *R*(*S*(*X*_*s*_)). Figure 4 shows the visualizations of the impacts of image reconstruction adversarial loss *L*_*advI*_ on *X*_*s*→*t*_. Finally, we train the segmentation network *S* with {(*X*_*s*→*t*_, *Y*_*s*_)} using following *L*_*trans*_ loss which is a combination of dice-coefficient loss and entropy minimization loss:


(7) $$\begin{array}{l} L_{dice}\left(X_{s\to t}\right)=1-\frac{2.Y_{s}^{\prime }.\tilde{Y_{s}^{\prime }}}{Y_{s}^{\prime }+\hat{Y_{s}^{\prime }}} \end{array}$$

(8) $$\begin{array}{l} L_{em}\left(X_{s\to t}\right)=-\frac{1}{H\times W}\underset{h,w}{\sum }\tilde{Y_{s}}\log\left(\tilde{Y_{s}}\right) \end{array}$$

(9) $$\begin{array}{l} L_{trans}\left(X_{s\to t}\right)=L_{dice}\left(X_{s\to t}\right)+L_{em}\left(X_{s\to t}\right) \end{array}$$

where $\tilde{Y_{s}}=S\left(X_{s\to t}\right)$. And, $Y_{s}^{\prime }$ and $\tilde{Y_{s}^{\prime }}$ are the flattened *Y*_*s*_ and $\tilde{Y_{s}}$, respectively.

#### 3.2.3. Discriminators

We utilize two discriminators in NuSegUDA: Prediction Discriminator (*D*_*P*_) and Image Discriminator (*D*_*I*_). Prediction Discriminator distinguishes between source domain outputs and target domain outputs, whereas Image Discriminator distinguishes between original images and reconstructed images. We discuss the details of both discriminators in the following.

**Prediction discriminator** As our goal is to generate similar predictions for both of source images and target images, we incorporate prediction discriminator *D*_*P*_ in NuSegUDA. This discriminator takes source domain prediction or target domain prediction as input, and then distinguishes whether the input (i.e., prediction) comes from the source domain or the target domain. To train *D*_*P*_, we use following cross-entropy loss:


(10) $$\begin{array}{l} L_{disP}(\hat{Y})\;=\;-\frac{1}{H_{p}\times W_{p}}\sum_{h_{p},w_{p}}z_{p}.\log(D_{P}(\hat{Y}))\\ \;+(1-z_{p}).\log(1-D_{P}(\hat{Y})) \end{array}$$

where *z*_*p*_=0 when *D*_*P*_ takes target domain prediction as its input, and *z*_*p*_=1 when the input comes from source domain prediction.

**Image discriminator** We use image discriminator *D*_*I*_ in NuSegUDA so that the reconstructed image distribution becomes similar to original image distribution. The input of *D*_*I*_ is either the original target image or the reconstructed target image. Then, *D*_*I*_ distinguishes whether the input is original or the reconstructed one. Similar to *D*_*P*_, we use following cross-entropy loss to train *D*_*I*_:


(11) $$\begin{array}{l} L_{disI}\left(X\right)=-\frac{1}{H_{i}\times W_{i}}\underset{h_{i},w_{i}}{\sum }z_{i}.\log\left(D_{I}\left(X\right)\right)+\left(1-z_{i}\right).\log\left(1-D_{I}\left(X\right)\right) \end{array}$$

where *z*_*i*_=0 when *D*_*I*_ takes reconstructed target image $\tilde{X_{t}}$ as its input, and *z*_*i*_=1 when the input comes from original target images *X*_*t*_.

#### 3.2.4. Feature-level adaptation

In addition to image-level domain adaptation at the outputs, we also apply feature-level domain adaptation in NuSegUDA to reduce the domain gap in the feature space. We assume that, our segmentation network *S* is composed of an encoder *S*_*E*_ and a decoder *S*_*D*_ (i.e., *S* = *S*_*E*_*oS*_*D*_). Here, the encoder *S*_*E*_ works as a feature extractor. Due to the discrepancy of input statistics across domains, there is also a shift of feature distribution in the feature space spanned by *S*_*E*_. Similar to Toldo et al. (2021), we utilize a clustering loss at the feature-level to serve as a constraint toward a class-conditional feature alignment between domains.

Given source image *X*_*s*_ and target image *X*_*t*_, we first extract the features *F*_*s*_ = *S*_*E*_(*X*_*s*_) and *F*_*t*_ = *S*_*E*_(*X*_*t*_). Then, the clustering loss is computed as:


(12) $$\begin{array}{l} L_{cl}(X_{s},X_{t})=\frac{1}{|F_{s,t}|}\sum_{f_{i}\in F_{s,t},{\hat{y}}_{i}\in {\hat{Y}}_{s,t}}d(f_{i},c_{{\hat{y}}_{i}})\\ \;-\frac{1}{|C|(|C|-1)}\sum_{j\in C}\sum_{k\in C,k\neq j}d(c_{j},c_{k}) \end{array}$$

where *f*_*i*_ is the feature vector corresponding to a spatial location of *F*_*s*_ or *F*_*t*_, ŷ_*i*_ is the corresponding predicted class, and *C* is the set of semantic classes which is {0, 1} for our nuclei segmentation problem. To compute ŷ_*i*_, the segmentation prediction Ŷ is downsampled to match the spatial dimension of *F*. We set the function *d*(.) to L1 norm. In Equation (12), *c*_*j*_ denotes the centroid of semantic class *j*, which is computed using following formula:


(13) $$c_{j}=\frac{\sum_{f_{i}}\sum_{{\hat{y}}_{i}}\delta_{j,{\hat{y}}_{i}}f_{i}}{\sum_{{\hat{y}}_{i}}\delta_{j,{\hat{y}}_{i}}},j\in \{0,1\}$$

where δ_*j*,_ŷ__*i*__ is equal to 1 if ŷ_*i*_ = *j*, and to 0 otherwise.

In Equation (12), the clustering loss is composed of two terms: the first term measures how close the features are from their respective centroids, and the second term measures how far the semantic class centroids are from each other. Therefore, according to the first term, the feature vectors of the same class from same or different domain are tightened around the class feature centroids. And, because of the second term, features from different classes gets a repulsive force applied to feature centroids which moves them apart.

Thus, we minimize the following total loss when training our segmentation network *S* and reconstruction network *R*:


(14) $$\begin{array}{l} L_{uda}(X_{s},X_{t})=L_{seg}(X_{s})+\lambda_{advP}L_{advP}(X_{t})+\lambda_{recons}L_{recons}(X_{t})+\\ \;\lambda_{advI}L_{advI}(X_{t})+\lambda_{trans}L_{trans}(X_{s\to t})+\lambda_{cl}L_{cl}(X_{s},X_{t}) \end{array}$$

where, λ_*advP*_, λ_*recons*_, λ_*advI*_, λ_*trans*_, and λ_*cl*_ are the weights to balance corresponding losses.

### 3.3. Semi-Supervised Domain Adaptation

In Semi-Supervised Domain Adaptation (SSDA) problem, we aims to ensure the best usages of available target domain annotations *Y*_*t*_ when training our segmentation network *S*. In such scenarios, we extend proposed NuSegUDA framework to NuSegSSDA, a nuclei segmentation SSDA model.

In NuSegSSDA, for unannotated target images $X_{t}^{u}$ we follow the same steps as NuSegUDA. However, when we encounter an annotated target data $\left(X_{t}^{l},Y_{t}\right)$ while training, we additionally compute the segmentation loss $L_{seg}\left(X_{t}^{l}\right)$ in the similar manner to Equation (3). Then, while computing the total loss we incorporate $L_{seg}\left(X_{t}^{l}\right)$ so that the segmentation network learns to generate the predictions closer to target ground-truths. Therefore, Equation (14) is now modified as below:


(15) $$\begin{array}{l} L_{ssda}(X_{s},X_{t}^{l},X_{t}^{u})=L_{seg}(X_{s})+L_{seg}(X_{t}^{l})+\lambda_{advP}L_{advP}(X_{t}^{u})\\ \;+\lambda_{recons}L_{recons}(X_{t}^{u})+\lambda_{advI}L_{advI}(X_{t}^{u})\\ \;+\lambda_{trans}L_{trans}(X_{s\to t})+\lambda_{cl}L_{cl}(X_{s},X_{t}^{l},X_{t}^{u}) \end{array}$$

## 4. Experiments

### 4.1. Datasets

In our experiments, we use two H&E stained histopathology datasets with ground-truth annotations for nuclei segmentation. Both of the datasets that we used are public. We present the brief of the datasets in the following.

**Dataset-1 (KIRC)** This dataset is taken from Irshad et al. (2014) in which the images are extracted at 40x magnification from Whole Slide Images (WSI) of Kidney Renal Clear cell carcinoma (KIRC). This dataset, referred as KIRC, consists of 486 H&E stained histology images of 400 × 400 pixel size with annotations made by expert pathologists and research fellows. In our experiments, we randomly split KIRC into 80% for training, 10% for validation, and 10% for testing.

**Dataset-2 (TNBC)** Naylor et al. (2018) generated this dataset by collecting slides from Triple Negative Breast Cancer (TNBC) patients at 40x magnification. For a total of 50 H&E stained histology images of pixel size 512 × 512, labeling was performed by expert pathologist and research fellows. We follow the same data splitting as KIRC for this dataset. We refer this dataset as TNBC in our experiments.

**Visual differences among datasets** Although both datasets consist of H&E stained histopathology images, they are collected from two different organs, cancer types, and institutions. KIRC images are collected from TCGA portal (image acquiring tools are unknown to us), whereas TNBC images were acquired at Curie Institute using Philips Ultra Fast Scanner 1.6RA. Organ difference, cancer type difference, institutional difference, and using different imaging tools and protocols cause the visual difference among the images from these two datasets. See Figure 2, where TNBC image looks dimmer than KIRC image.

### 4.2. Implementations

In our work, we use U-Net (Ronneberger et al., 2015) as both of our segmentation network and reconstruction network. We choose U-Net so that our proposed segmentation framework can be directly applied in other biomedical domains. We preferred U-Net over UNet++ (Zhou et al., 2018) because of the less number of parameters. Following DCGAN (Radford et al., 2015), we designed our prediction discriminator and image discriminator consisting of five convolutional layers. To train NuSegUDA and NuSegSSDA, we followed the training strategy from GAN (Goodfellow et al., 2014). Adam optimizer (Kingma and Ba, 2014) with learning rate 0.0001, 0.001, 0.001, and 0.001 are used in segmentation network, reconstruction network, prediction discriminator, and image discriminator, respectively. We empirically choose 0.001, 0.01, 0.001, 0.001, and 0.002 as λ_*advP*_, λ_*recons*_, λ_*advI*_, λ_*trans*_, and λ_*cl*_, respectively. We implement NuSegUDA and NuSegSSDA using PyTorch (Paszke et al., 2019), and trained on a single GPU. We do not use any data augmentation in our experiments.

### 4.3. Experimental results

#### 4.3.1. Unsupervised Domain Adaptation

**Experiment-1 (KIRC → TNBC)** In our first experiment, we choose KIRC as source domain and TNBC as target domain, denoted by KIRC → TNBC. In our experiment, we choose U-Net (Ronneberger et al., 2015) as the representative of Convolutional Neural Network (CNN) based approaches. Fully-supervised segmentation model U-Net gives an insight of how it performs when directly applying transfer learning (i.e., training with only KIRC and then test it on TNBC without any modifications). AdaptSegNet (Tsai et al., 2018) and OrClEmb (Toldo et al., 2021) represent generic Unsupervised Domain Adaptation (UDA) models. DA-ADV (Dong et al., 2018), CellSegUDA (Haq and Huang, 2020), EndoUDA (Celik et al., 2021), SelfEnsemb (Li et al., 2021), and MaNi (Sharma et al., 2022) are chosen as the representatives of UDA model for biomedical image segmentation.

**Figure 5:**
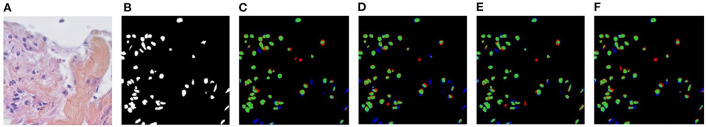
Visualizations of Unsupervised Domain Adaptation (UDA) for KIRC → TNBC. **(A)** Target image, **(B)** Ground-truth, **(C)** CellSegUDA, **(D)** SelfEnsemb, **(E)** MaNi, and **(F)** NuSegUDA (ours). In **(C–F)**, green pixels, red pixels, and blue pixels indicate the true positives, false positives, and false negatives, respectively. In other words, green and red pixels indicate the predicted nuclei pixels, whereas green and blue pixels indicate the ground-truth nuclei pixels. This average-dense nuclei histopathology image in **(A)** is chosen so that the reader can easily find out the visual differences without further zooming-in.

From Table 1, we see that source-trained U-Net gives the lower-bound of experimental performance (see first row of Table 1) which happens because of the visual domain gap between source training images and target test images, also known as domain shift problem. We see that, our proposed UDA model NuSegUDA outperforms all UDA baseline models in terms of IoU%, Dice score, and Hausdorff distance. Specifically, NuSegUDA has 1.28 and 0.42 higher IoU% than best generic UDA baseline OrClEmb, and best biomedical UDA baseline MaNi, respectively. Figure 5 shows the visualization results of CellSegUDA, SelfEnsemb, MaNi, and NuSegUDA. In Table 1, the second to last row [i.e., U-Net (target-trained)] shows the upper-bound of experimental performance (i.e., training U-Net with TNBC-train and testing it on TNBC-test).

**Table 1:** Baseline sociodemographic characteristics of participants in the study.

	**Experiment-1**			**Experiment-2**		
	**KIRC**→**TNBC**			**TNBC**→**KIRC**		
**Method**	**IoU%**	**Dice score**	**HD**	**IoU%**	**Dice score**	**HD**
U-Net (source-trained)	52.66	0.6875	10.1214	54.82	0.7056	9.2487
DA-ADV	54.93	0.7079	9.6531	55.43	0.7107	9.0142
AdaptSegNet	56.49	0.7198	9.1512	56.87	0.7235	8.3477
CellSegUDA	59.02	0.7394	8.5653	57.09	0.7242	8.1739
OrClEmb	59.23	0.7402	8.5564	57.05	0.7236	8.1923
EndoUDA	59.81	0.7445	8.3317	57.39	0.7277	8.1254
SelfEnsemb	60.02	0.7468	8.2524	57.45	0.7292	8.1121
MaNi	60.09	0.7477	8.2746	57.48	0.7293	8.1493
U-Net (target-trained)	66.57	0.7985	7.7301	62.04	0.7621	7.6281
NuSegUDA (ours)	60.51	0.7525	8.0011	57.68	0.7303	8.0881

Unsupervised Domain Adaptation (UDA) results for Experiment-1 and Experiment-2. IoU and HD denotes Intersection over Union, and Hausdorff distance, respectively. Results are from testing on TNBC-test and KIRC-test for experiment-1 and experiment-2, respectively.

**Experiment-2 (TNBC → KIRC)** We conduct another experiment in the similar way to experiment-1 by selecting TNBC as source and KIRC as target domain. This experiment also reflects the excellence of NuSegUDA compared to other approaches in terms of segmentation accuracies (see last three columns of Table 1).

#### 4.3.2. Semi-Supervised Domain Adaptation

**Figure 6:**
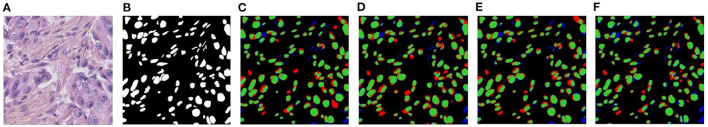
Visualizations of Semi-Supervised Domain Adaptation (SSDA) for KIRC → TNBC. **(A)** Target image, **(B)** Ground-truth, **(C)** NuSegSSDA (10%), **(D)** NuSegSSDA (25%), **(E)** NuSegSSDA (50%), and **(F)** NuSegSSDA (75%). In **(C-F)**, green pixels, red pixels, and blue pixels indicate the true positives, false positives, and false negatives, respectively.

**Experiment-1 (KIRC → TNBC)** In experiment-1, we assess our Semi-Supervised Domain Adaptation (SSDA) method NuSegSSDA for KIRC → TNBC. Table 2 shows the experimental performances of NuSegSSDA. For this experiment, the source dataset KIRC is the same as UDA experiments. However, now we treat TNBC as partially labeled. We train NuSegSSDA considering 10%, 25%, 50%, and 75% images from TNBC-train dataset have annotations available. Then, testing on TNBC-test gives us increasing IoUs and Dice scores, and decreasing Hausdorff Distances. This happens because more false negative nuclei can be identified and some false positive nuclei can be removed by NuSegSSDA as we train it with more target annotations (see Figure 6). We compare NuSegSSDA with fully-supervised model U-Net (Ronneberger et al., 2015), and baseline biomedical SSDA model CellSegSSDA (Haq and Huang, 2020) to demonstrate the superiority of our proposed SSDA model. To train U-Net, we combine full KIRC dataset with the same 10%, 25%, 50%, and 75% of TNBC-train we chose to train NuSegSSDA. We observe that, the accuracy of NuSegSSDA approaches to the upper-bound (only lower by 1.35 IoU%) as we train with more annotations from target domain.

**Table 2:** Semi-Supervised Domain Adaptation (SSDA) results for Experiment-1 and Experiment-2.

	**Experiment-1**			**Experiment-2**		
	**KIRC**→**TNBC**			**TNBC**→**KIRC**		
**Method**	**IoU%**	**Dice**	**HD**	**IoU%**	**Dice**	**HD**
U-Net (source 100% + target 10%)	60.74	0.7534	8.3627	56.89	0.7194	8.5122
CellSegSSDA (source 100% + target 10%)	60.96	0.7557	8.3563	58.81	0.7377	7.9817
NuSegSSDA (source 100% + target 10%) (ours)	**61.12**	**0.7578**	**8.3274**	**58.99**	**0.7401**	**7.9629**
U-Net (source 100% + target 25%)	61.67	0.7607	8.2742	59.32	0.7405	7.9211
CellSegSSDA (source 100% + target 25%)	62.94	0.771	8.0966	59.73	0.7443	**7.8647**
NuSegSSDA (source 100% + target 25%) (ours)	**63.15**	**0.7732**	**8.0487**	**59.79**	**0.7449**	7.8752
U-Net (source 100% + target 50%)	56.73	0.7208	9.1473	59.95	0.7464	7.8461
CellSegSSDA (source 100% + target 50%)	63.59	0.7748	7.9802	60.32	0.7494	7.7958
NuSegSSDA (source 100% + target 50%) (ours)	**63.97**	**0.7802**	**7.9549**	**60.53**	**0.7511**	**7.7754**
U-Net (source 100% + target 75%)	59.06	0.7394	8.6286	61.63	0.7592	7.7026
CellSegSSDA (source 100% + target 75%)	64.96	0.7862	7.8496	61.01	0.7541	7.7275
NuSegSSDA (source 100% + target 75%) (ours)	**65.22**	**0.7901**	**7.7928**	**61.68**	**0.7598**	**7.6872**
U-Net (target 100%)	66.57	0.7985	7.7301	62.04	0.7621	7.6281

IoU, Dice, and HD denotes Intersection over Union, Dice score, and Hausdorff Distance, respectively. NuSegSSDA refers to our proposed SSDA model. NuSegSSDA (source 100% + target *n*%) denotes *n*% annotations available in TNBC-train and KIRC-train for experiment-1 and experiment-2, respectively. Results are from testing on TNBC-test and KIRC-test for experiment-1 and experiment-2, respectively. Bold values denote the best scores among the experiments for different *n* percentages (i.e., source 100% + target *n*%).

**Experiment-2 (TNBC → KIRC)** In our second experiment, we select TNBC as source and KIRC as target domain. The second experiment also demonstrates the excellence of NuSegSSDA compared to U-Net (Ronneberger et al., 2015) and CellSegSSDA (Haq and Huang, 2020) (see last three columns of Table 2). Similar to experiment-1, for the second experiment we again see that the segmentation accuracies of NuSegSSDA increase when more target images are annotated.

#### 4.3.3. Ablation studies

To verify the robustness of proposed UDA framework, we perform extensive ablation studies on the adaptation of NuSegUDA from KIRC to TNBC, and from TNBC to KIRC. First, we examine the contribution of each loss to the final IoU%, Dice score, and Hausdorff Distance; then, we investigate the effects of different segmentation network backbones on NuSegUDA.

**Effectiveness of losses** The contribution of image adversarial loss *L*_*advI*_, target-translated source supervised loss *L*_*trans*_, and clustering loss *L*_*cl*_ to our proposed NuSegUDA model is shown in Table 3. We see that, simply applying only *L*_*advI*_ or *L*_*cl*_ to CellSegUDA (Haq and Huang, 2020) gives little better performance than CellSegUDA alone. However, when we apply only target-translated source supervised loss *L*_*trans*_ to CellSegUDA, the performance is inferior due to the absence of *L*_*advI*_ loss. Without applying image-adversarial loss *L*_*advI*_, target-translated source images *X*_*s*→*t*_ looks very different from the target-domain images in terms of texture, style, color distribution, etc. (see Figure 4). As a result, the performance of the model (i.e., CellSegUDA w/ *L*_*trans*_) decreases when trained with these *X*_*s*→*t*_ images.

**Table 3:** Impacts of *L*_*advI*_, *L*_*trans*_, and *L*_*cl*_ loss on NuSegUDA for Experiment-1 and Experiment-2.

				**Experiment-1**			**Experiment-2**		
				**KIRC**→**TNBC**			**TNBC**→**KIRC**		
**Method**	*L*_*advI*_	*L*_*trans*_	*L*_*cl*_	**IoU%**	**Dice**	**HD**	**IoU%**	**Dice**	**HD**
CellSegUDA				59.02	0.7394	8.5653	57.09	0.7242	8.1739
CellSegUDA w/ *L*_*advI*_	✓			59.38	0.7405	8.4316	57.17	0.7252	8.1422
CellSegUDA w/ *L*_*trans*_		✓		58.44	0.7357	8.6123	56.77	0.7209	8.3865
CellSegUDA w/ *L*_*cl*_			✓	59.11	0.7398	8.5734	57.02	0.7237	8.1203
NuSegUDA w/o *L*_*advI*_		✓	✓	58.59	0.7365	8.5914	56.82	0.7212	8.3685
NuSegUDA w/o *L*_*trans*_	✓		✓	59.45	0.7411	8.4021	57.19	0.7253	8.2468
NuSegUDA w/o *L*_*cl*_	✓	✓		60.36	0.7512	8.1963	57.63	0.7298	8.1247
NuSegUDA (ours)	✓	✓	✓	60.51	0.7525	8.0011	57.68	0.7303	8.0880

IoU, Dice, and HD denotes Intersection over Union, Dice score, and Hausdorff Distance, respectively. NuSegUDA w/o *L*_*advI*_, NuSegUDA w/o *L*_*trans*_, and NuSegUDA w/o *L*_*cl*_ refer to our proposed UDA model without image adversarial loss, target-translated source supervised loss, and clustering loss, respectively. Results are from testing on TNBC-test and KIRC-test for experiment-1 and experiment-2, respectively.

**Figure 7:**
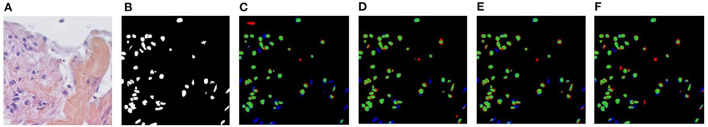
Visualizations of the effectiveness of proposed *L*_*advI*_, *L*_*trans*_, and *L*_*cl*_ loss on NuSegUDA for KIRC → TNBC. **(A)** Target image, **(B)** Ground-truth, **(C)** NuSegUDA w/o *L*_*advl*_, **(D)** NuSegUDA w/o *L*_*trans*_, **(E)** NuSegUDA w/o *L*_*cl*_, and **(F)** NuSegUDA. In **(C-F)**, green pixels, red pixels, and blue pixels indicate the true positives, false positives, and false negatives, respectively.

Similarly, NuSegUDA w/o *L*_*advI*_ gives much worse performance than NuSegUDA which happens because of training NuSegUDA with less-realistic target-translated source domain images. This again validates the effectiveness of *L*_*advI*_ on NuSegUDA. Finally, with all the proposed losses enabled, we achieve the best performing model NuSegUDA for both of the experiments which demonstrates the combined impact of newly proposed image adversarial loss, target-translated source supervised loss, and clustering loss on NuSegUDA. Figure 7 shows the visualization results of NuSegUDA w/o *L*_*advI*_, NuSegUDA w/o *L*_*trans*_, NuSegUDA w/o *L*_*cl*_, and NuSegUDA.

**Impacts of different segmentation networks** In NuSegUDA, we use U-Net (Ronneberger et al., 2015) as the backbone segmentation network. We also assess the model performance by replacing the backbone segmentation network with two more frequently-used Convolutional Neural Network (CNN) based approaches: FCN (Long et al., 2015) and UNet++ (Zhou et al., 2018). As mentioned earlier, CNN based approaches are still the dominant ones for semantic segmentation of nuclei. However, due to the intrinsic locality nature and limited receptive fields of convolution operations, CNN based models may be incapable of capturing the global context of the input (Chen et al., 2021; Jia et al., 2021; Zheng et al., 2021). To this end, we explore the feasibility of Transformers, an alternative to CNNs, as the backbone segmentation network in NuSegUDA. Transformer mainly utilizes self-attention mechanism to extract inherent features (Tay et al., 2020), and due to this self-attention mechanism, transformers are powerful at modeling the global context of an input (Zheng et al., 2021). To examine the effectiveness of Vision Transformer based model, we replace U-Net in NuSegUDA with TransUNet (Chen et al., 2021) which basically combines a hybrid CNN-transformer encoder architecture with a decoder.

Table 4 shows the quantitative results of using different segmentation networks in NuSegUDA. We see that, among CNN-based models, UNet++ and U-Net outperform other CNN approaches in Experiment-1, and Experiment-2, respectively. We also see that, Transformer-based model TransUNet does not give any better accuracy than U-Net and UNet++ for both of the experiments. This happens due to our small-sized training datasets, because Vision Transformers (VT) need lot of data for training, usually more than what is necessary to standard CNNs (Liu et al., 2021).

**Table 4:** Impacts of different segmentation network backbones in NuSegUDA.

**Segmentation network**	**Experiment-1**			**Experiment-2**		
	**KIRC**→**TNBC**			**TNBC**→**KIRC**		

	**IoU%**	**Dice score**	**HD**	**IoU%**	**Dice score**	**HD**
FCN	59.23	0.7398	8.4125	55.81	0.7165	8.7365
U-Net	60.51	0.7525	8.0011	57.68	0.7303	8.0880
UNet++	60.57	0.7529	8.0336	57.41	0.7282	8.1575
TransUNet	59.87	0.7476	8.1562	57.02	0.7256	8.1742

## 5. Conclusion

Accurate nuclei segmentation is a significant step for cancer diagnosis and further clinical procedures. Collecting a fully annotated nuclei segmentation dataset, or manually labeling an unannotated dataset is expensive, time-consuming, and impractical although such annotations are required to train Convolutional Neural Networks in fully-supervised manner. In this work, we propose a novel Unsupervised Domain Adaptation (UDA) framework named NuSegUDA for segmenting nuclei in unannotated datasets by utilizing adversarial learning. In NuSegUDA, we apply domain adaptation at both of feature space and output space. We also incorporate image adversarial loss and target-translated source supervised loss into NuSegUDA, and train the model with target-translated source domain images. Extensive and prominent experimental results validate the effectiveness of each of the newly proposed modules and losses, and the superiority of NuSegUDA over baseline models. Finally, assuming we have a few annotations available, we extend our work to Semi-Supervised Domain Adaptation (SSDA). We expect our proposed UDA and SSDA approaches to be very useful in other biomedical image segmentation tasks.

## Data availability statement

The original contributions presented in the study are included in the article/supplementary material, further inquiries can be directed to the corresponding author.

## Author contributions

MH, HM, and JH contributed to the methodology and design of the study. MH implemented the proposed method, organized the experimental section, and wrote the first draft of the manuscript. HM and JH corrected the draft. All authors contributed to the article and approved the submitted version.

## References

[B1] Boykov Y. Kolmogorov V. (2004). An experimental comparison of min-cut/max-flow algorithms for energy minimization in vision. IEEE Trans. Pattern Anal. Mach. Intell. 26, 1124–1137. 10.1109/TPAMI.2004.60 15742889

[B2] Celik N. Ali S. Gupta S. Braden B. Rittscher J. (2021). “Endouda: a modality independent segmentation approach for endoscopy imaging,” in International Conference on Medical Image Computing and Computer-Assisted Intervention - MICCAI 2021. Lecture Notes in Computer Science, Vol. 12903 (Cham: Springer), 303–312. 10.1007/978-3-030-87199-4_29

[B3] Chen C. Dou Q. Chen H. Qin J. Heng P.-A. (2019). “Synergistic image and feature adaptation: towards cross-modality domain adaptation for medical image segmentation,” in AAAI'19: Proceedings of the AAAI Conference on Artificial Intelligence, Vol. 33 (Honolulu, HI), 865–872. 10.1609/aaai.v33i01.3301865

[B4] Chen J. Lu Y. Yu Q. Luo X. Adeli E. Wang Y.. (2021). TransUNet: Transformers make strong encoders for medical image segmentation. arXiv Preprint arXiv:2102.04306. 10.48550/arXiv.2102.04306

[B5] Chen Y.-C. Lin Y.-Y. Yang M.-H. Huang J.-B. (2019). “CrDoCo: Pixel-level domain transfer with cross-domain consistency,” in 2019 Proceedings of the IEEE Conference on Computer Vision and Pattern Recognition (Long Beach, CA), 1791–1800. 10.1109/CVPR.2019.00189

[B6] Dong N. Kampffmeyer M. Liang X. Wang Z. Dai W. Xing E. (2018). “Unsupervised domain adaptation for automatic estimation of cardiothoracic ratio,” in International Conference on Medical Image Computing and Computer-Assisted Intervention - MICCAI 2018. Lecture Notes in Computer Science, Vol. 11071, eds A. Frangi, J. Schnabel, C. Davatzikos, C. Alberola-López, and G. Fichtinger (Cham: Springer), 544–552. 10.1007/978-3-030-00934-2_61

[B7] Gholami A. Subramanian S. Shenoy V. Himthani N. Yue X. Zhao S.. (2019). “A novel domain adaptation framework for medical image segmentation,” in International MICCAI Brainlesion Workshop. BrainLes 2018: Brainlesion: Glioma, Multiple Sclerosis, Stroke and Traumatic Brain Injuries. BrainLes 2018. Lecture Notes in Computer Science, Vol. 11384, eds A. Crimi, S. Bakas, H. Kuijf, F. Keyvan, M. Reyes, and T. van Walsum (Cham: Springer), 289–298. 10.1007/978-3-030-11726-9_26

[B8] Goodfellow I. Pouget-Abadie J. Mirza M. Xu B. Warde-Farley D. Ozair S.. (2014). “Generative adversarial nets,” in Advances in Neural Information Processing Systems 27 (NIPS 2014), eds Z. Ghahramani, M. Welling, C. Cortes, N. Lawrence, and K. Q. Weinberger (Montreal, QC), 2672–2680.

[B9] Haq M. M. Huang J. (2020). “Adversarial domain adaptation for cell segmentation,” in Proceedings of the Third Conference on Medical Imaging with Deep Learning, PMLR, Vol. 121 eds T. Arbel, I. B. Ayed, M. de Bruijne, M. Descoteaux, H. Lombaert, and C. Pal (Montreal, QC), 277–287.

[B10] Haq M. M. Huang J. (2022). “Self-supervised pre-training for nuclei segmentation,” in International Conference on Medical Image Computing and Computer-Assisted Intervention. Medical Image Computing and Computer Assisted Intervention - MICCAI 2022. Lecture Notes in Computer Science, Vol. 13432, eds L. Wang, Q. Dou, P.T. Fletcher, S. Speidel, and S. Li (Cham: Springer), 303–313. 10.1007/978-3-031-16434-7_30

[B11] Hoffman J. Tzeng E. Park T. Zhu J.-Y. Isola P. Saenko K.. (2017). Cycada: Cycle-consistent adversarial domain adaptation. arXiv Preprint arXiv:1711.03213. 10.48550/arXiv.1711.03213

[B12] Hou L. Agarwal A. Samaras D. Kurc T. M. Gupta R. R. Saltz J. H. (2019). “Robust histopathology image analysis: to label or to synthesize?,” in Proceedings of the IEEE Conference on Computer Vision and Pattern Recognition (Long Beach, CA), 8533–8542. 10.1109/CVPR.2019.00873 PMC8139403 34025103

[B13] Huo Y. Xu Z. Bao S. Assad A. Abramson R. G. Landman B. A. (2018). “Adversarial synthesis learning enables segmentation without target modality ground truth,” in 2018 IEEE 15th International Symposium on Biomedical Imaging (ISBI 2018) (Washington, DC: IEEE), 1217–1220. 10.1109/ISBI.2018.8363790 PMC12462902 41018946

[B14] Irshad H. Montaser-Kouhsari L. Waltz G. Bucur O. Nowak J. Dong F.. (2014). Crowdsourcing image annotation for nucleus detection and segmentation in computational pathology: evaluating experts, automated methods, and the crowd. Pac. Symp. Biocomput. 2015, 294–305. 10.1142/9789814644730_0029 25592590 PMC4299942

[B15] Isola P. Zhu J.-Y. Zhou T. Efros A. A. (2017). “Image-to-image translation with conditional adversarial networks,” in Proceedings of the IEEE Conference on Computer Vision and Pattern Recognition (Honolulu, HI), 1125–1134. 10.1109/CVPR.2017.632

[B16] Jia H. Tang H. Ma G. Cai W. Huang H. Zhan L.. (2021). PSGR: Pixel-wise sparse graph reasoning for COVID-19 pneumonia segmentation in CT images. arXiv Preprint. arXiv:2108.03809. 10.48550/arXiv.2108.03809 PMC9942482 36842219

[B17] Kamnitsas K. Baumgartner C. Ledig C. Newcombe V. Simpson J. Kane A.. (2017). “Unsupervised domain adaptation in brain lesion segmentation with adversarial networks,” in International Conference on Information Processing in Medical Imaging. IPMI 2017. Lecture Notes in Computer Science, Vol. 10265 (Cham: Springer), 597–609. 10.1007/978-3-319-59050-9_47

[B18] Kingma D. P. Ba J. (2014). Adam: A method for stochastic optimization. arXiv Preprint. arXiv:1412.6980. 10.48550/arXiv.1412.6980

[B19] Kumar N. Verma R. Sharma S. Bhargava S. Vahadane A. Sethi A. (2017). A dataset and a technique for generalized nuclear segmentation for computational pathology. IEEE Trans. Med. Imaging 36, 1550–1560. 10.1109/TMI.2017.2677499 28287963

[B20] Li C. Zhou Y. Shi T. Wu Y. Yang M. Li Z. (2021). “Unsupervised domain adaptation for the histopathological cell segmentation through self-ensembling,” in Proceedings of the MICCAI Workshop on Computational Pathology, PMLR 156, 151–158. Available online at: https://proceedings.mlr.press/v156/li21a.html

[B21] Liu Y. Sangineto E. Bi W. Sebe N. Lepri B. Nadai M. (2021). “Efficient training of visual transformers with small datasets,” in Proceedings of the 35th Conference on Neural Information Processing Systems (NeurIPS) 2021 (Virtual).

[B22] Long J. Shelhamer E. Darrell T. (2015). “Fully convolutional networks for semantic segmentation,” in Proceedings of the IEEE Conference on Computer Vision and Pattern Recognition (Boston, MA), 3431–3440. 10.1109/CVPR.2015.7298965 27244717

[B23] Mahmood F. Borders D. Chen R. J. McKay G. N. Salimian K. J. Baras A.. (2019). Deep adversarial training for multi-organ nuclei segmentation in histopathology images. IEEE Trans. Med. Imaging 39, 3257–3267. 10.1109/TMI.2019.2927182 31283474 PMC8588951

[B24] Mirza M. Osindero S. (2014). Conditional generative adversarial nets. arXiv Preprint arXiv:1411.1784. 10.48550/arXiv.1411.1784

[B25] Naylor P. Laé M. Reyal F. Walter T. (2018). Segmentation of nuclei in histopathology images by deep regression of the distance map. IEEE Trans. Med. Imaging 38, 448–459. 10.1109/TMI.2018.2865709 30716022

[B26] Paszke A. Gross S. Massa F. Lerer A. Bradbury J. Chanan G.. (2019). Pytorch: An imperative style, high-performance deep learning library. NIPS'19: Proceedings of the 33rd International Conference on Neural Information Processing Systems 32 (Vancouver, BC), 8026–8037.

[B27] Radford A. Metz L. Chintala S. (2015). Unsupervised representation learning with deep convolutional generative adversarial networks. arXiv Preprint. arXiv:1511.06434. 10.48550/arXiv.1511.06434

[B28] Raju A. Ji Z. Cheng C. T. Cai J. Huang J. Xiao J.. (2020). “User-guided domain adaptation for rapid annotation from user interactions: a study on pathological liver segmentation,” in International Conference on Medical Image Computing and Computer-Assisted Intervention - MICCAI 2020. Lecture Notes in Computer Science, Vol. 12261 (Cham: Springer), 457–467. 10.1007/978-3-030-59710-8_45

[B29] Ronneberger O. Fischer P. Brox T. (2015). “U-net: Convolutional networks for biomedical image segmentation,” in International Conference on Medical Image Computing and Computer-Assisted Intervention - MICCAI 2015. Lecture Notes in Computer Science, Vol. 9351 (Cham: Springer), 234–241. 10.1007/978-3-319-24574-4_28

[B30] Sharma Y. Syed S. Brown D. E. (2022). “Mani: Maximizing mutual information for nuclei cross-domain unsupervised segmentation,” in International Conference on Medical Image Computing and Computer-Assisted Intervention, eds L. Wang, Q. Dou, P.T. Fletcher, S. Speidel, and S. Li (Cham: Springer), 345–355. 10.1007/978-3-031-16434-7_34

[B31] Sirinukunwattana K. Raza S. E. A. Tsang Y.-W. Snead D. R. Cree I. A. Rajpoot N. M. (2016). Locality sensitive deep learning for detection and classification of nuclei in routine colon cancer histology images. IEEE Trans. Med. Imaging 35, 1196–1206. 10.1109/TMI.2016.2525803 26863654

[B32] Tay Y. Dehghani M. Bahri D. Metzler D. (2020). Efficient transformers: a survey. arXiv Preprint arXiv:2009.06732. 10.48550/arXiv.2009.06732

[B33] Toldo M. Michieli U. Zanuttigh P. (2021). “Unsupervised domain adaptation in semantic segmentation via orthogonal and clustered embeddings,” in 2021 Proceedings of the IEEE/CVF Winter Conference on Applications of Computer Vision (Waikoloa, HI), 1358–1368. 10.1109/WACV48630.2021.00140

[B34] Tsai Y.-H. Hung W.-C. Schulter S. Sohn K. Yang M.-H. Chandraker M. (2018). “Learning to adapt structured output space for semantic segmentation,” in 2018 Proceedings of the IEEE Conference on Computer Vision and Pattern Recognition (Salt Lake City, UT), 7472–7481. 10.1109/CVPR.2018.00780

[B35] Xia X. Kulis B. (2017). W-net: A deep model for fully unsupervised image segmentation. arXiv Preprint. arXiv:1711.08506. 10.48550/arXiv.1711.0850634460779

[B36] Xia Y. Yang D. Yu Z. Liu F. Cai J. Yu L.. (2020). Uncertainty-aware multi-view co-training for semi-supervised medical image segmentation and domain adaptation. Med. Image Anal. 65, 101766. 10.1016/j.media.2020.101766 32623276

[B37] Xu Y. Jia Z. Wang L.-B. Ai Y. Zhang F. Lai M.. (2017). Large scale tissue histopathology image classification, segmentation, and visualization via deep convolutional activation features. BMC Bioinformatics 18:281. 10.1186/s12859-017-1685-x 28549410 PMC5446756

[B38] Yang J. Li C. An W. Ma H. Guo Y. Rong Y.. (2021). “Exploring robustness of unsupervised domain adaptation in semantic segmentation,” in Proceedings of the IEEE/CVF International Conference on Computer Vision (Montreal, BC), 9194–9203. 10.1109/ICCV48922.2021.00906

[B39] Yang S. Zhang J. Huang J. Lovell B. C. Han X. (2021). “Minimizing labeling cost for nuclei instance segmentation and classification with cross-domain images and weak labels,” in Proceedings of the AAAI Conference on Artificial Intelligence, Vol. 35 (Virtual), 697–705. 10.1609/aaai.v35i1.16150

[B40] Zeiler M. D. Fergus R. (2014). “Visualizing and understanding convolutional networks,” in European Conference on Computer Vision - ECCV 2014. Lecture Notes in Computer Science, eds D. Fleet, T. Pajdla, B. Schiele, and T. Tuytelaars (Cham: Springer), 818–833. 10.1007/978-3-319-10590-1_53

[B41] Zhang Y. Qiu Z. Yao T. Liu D. Mei T. (2018). “Fully convolutional adaptation networks for semantic segmentation,” in Proceedings of the IEEE Conference on Computer Vision and Pattern Recognition (Salt Lake City, UT), 6810–6818. 10.1109/CVPR.2018.00712

[B42] Zheng S. Lu J. Zhao H. Zhu X. Luo Z. Wang Y.. (2021). “Rethinking semantic segmentation from a sequence-to-sequence perspective with transformers,” in Proceedings of the IEEE/CVF Conference on Computer Vision and Pattern Recognition (Nashville, TN), 6881–6890. 10.1109/CVPR46437.2021.00681

[B43] Zhou Z. Siddiquee M. M. R. Tajbakhsh N. Liang J. (2018). “UNet++: A nested u-net architecture for medical image segmentation,” in Deep Learning in Medical Image Analysis and Multimodal Learning for Clinical Decision Support. DLMIA ML-CDS 2018. Lecture Notes in Computer Science, Vol. 11045 (Cham: Springer), 3–11. 10.1007/978-3-030-00889-5_1 PMC7329239 32613207

[B44] Zhu J.-Y. Park T. Isola P. Efros A. A. (2017). “Unpaired image-to-image translation using cycle-consistent adversarial networks,” in Proceedings of the IEEE International Conference on Computer Vision (ICCV 2017) (Venice), 2223–2232. 10.1109/ICCV.2017.244

